# Trends in Lakeshore Zone Development: A Comparison of Polish and Hungarian Lakes over 30-Year Period

**DOI:** 10.3390/ijerph19042141

**Published:** 2022-02-14

**Authors:** Grażyna Furgała-Selezniow, Małgorzata Jankun-Woźnicka, Paweł Woźnicki, Xuecheng Cai, Timea Erdei, Zsombor Boromisza

**Affiliations:** 1 Department of Tourism, Recreation and Ecology, University of Warmia and Mazury in Olsztyn, Oczapowskiego 5, 10-718 Olsztyn, Poland; mjpw@uwm.edu.pl; 2 Independent Researcher, 10-098 Olsztyn, Poland; polypterus9@gmail.com; 3 Department of Landscape Protection and Restoration, Hungarian University of Agriculture and Life Sciences, Villányi út 35-43, 1118 Budapest, Hungary; chercaicai@gmail.com (X.C.); erdeitimi@gmail.com (T.E.); boromisza.zsombor@uni-mate.hu (Z.B.)

**Keywords:** LU/LC change, sustainable land use, water recreation infrastructure expansion, water-based tourism development

## Abstract

(1) Background: This paper presents the land use and land cover change processes in the lakeshore zone in Poland and Hungary during 30 years. (2) Methods: Land use and land cover (LU/LC) maps were prepared using topographic maps and orthophotograph maps scaled 1:10,000. The study based on GIS data and field research. (3) Results: A significant increase in the area occupied by tourist and recreational infrastructure and forests in the lake shore zone was found in both countries. In Poland, this increase occurred mainly at the expense of arable land, which was a positive phenomenon. In Hungary, however, the main threat to the lakeshore zone was the increase of built-up area at the expense of semi-natural area. While the decrease in arable land was positive from an environmental point of view, the main threat to the Hungarian lake shore zone was the increase in built-up areas at the expense of semi-natural land. The results showed a positive correlation between the area of urbanized land and the area occupied by tourist and recreational buildings on the Polish lakes. There was no such correlation at the Hungarian lakes. (4) Conclusions: The most beneficial change in land cover for the lakes was the increase in forest area in the lake shore zone in both countries studied. Taking into account the results of previous studies, three main trends of changes in land cover and land use in the lakeshore zone were identified. These results shed new light on the problem of land use around lakeshores.

## 1. Introduction

Land use change has accompanied humankind since its beginnings and is inextricably linked to its current and future development [1,2]. Land use (LU) and land cover (LC) are the two basic elements that describe changes caused by human transformation of the terrestrial environment [3,4]. Until the early 19th century, these changes were mainly local. After the Industrial Revolution, these changes began to take on a global dimension. The rapid escalation of this process occurred in the second half of the 20th century (after World War II) and continues to the present. The increase in the world’s human population (from about 2 billion to more than 7 billion in the aforementioned period) results in increasing demands for food, energy, and living space [5,6]. These growing needs generate changes in land cover, which has a negative impact on the state of the natural environment. They particularly affect climate, ecosystem services, water resources, and biodiversity [7,8,9], and consequently affect human well-being [10]. 

Land use is often determined by social and economic conditions in a region. If these conditions change radically and rapidly, previous land use rules may become obsolete [11]. A good example of such a situation was the collapse of socialism in Central and Eastern European countries [12,13]. The change of regime in eastern and central Europe resulted in deep changes in agriculture. Above all, the area of land occupied by crops decreased substantially [14,15]. In Poland, unlike in other countries of the region, most of the land used for agriculture (more than four fifths) was in the hands of private smallholders [12]. After 1989, the economic viability of agricultural production declined. According to Łowicki, between 1990 and 2004, Poland experienced a larger decrease in agricultural land area than in the period from 1938 to 1990 [16]. Social and economic transformations in Poland have also had a significant impact on the tourism industry. In rural areas, tourism has become an important source of additional income. In the agricultural areas nationalized in Hungary after 1945, collective farms have been running the economy since the 1960s. Land that was not suitable for large-scale agricultural production was divided into smaller plots, where vineyards, orchards or vegetable gardens were established. Privatization, which began in the 1990s, has contributed to a decline in agricultural land of around 1.2 million hectares [17]. The rate of decline in agricultural land was more than 17%, while the size of land taken out of cultivation increased by nearly 80%. The number of individual farms has decreased significantly, while large-scale farms have emerged, which is characteristic of countries that have experienced agrarian collectivism [18]. Large-scale farming represents the ‘industrial type’ of production in the Hungarian landscape. Next to large-scale crops, there are abandoned agricultural areas, overgrown with invasive species or showing natural succession processes. These are mainly former small cultivated plots, some of which have also become residential or recreational areas [17]. Another important factor affecting agricultural areas is climate change. For example, the efficiency of crops in areas plagued by drought decreases, and so does their profitability. This phenomenon leads to the abandonment of crops in these areas, resulting in a reduction in the area of agricultural land [19].

Lakes, as bodies of fresh water, are of great importance to both the natural environment and humans. Archaeological data show that the process of settlement around lake shores has been a long known phenomenon [20]. The second half of the 20th century saw a significant development of lake-related tourism and recreation [21]. The lakeshore zone is a special area—on the one hand, very attractive for tourism, on the other hand, extremely vulnerable to negative aspects of human activities [22,23]. Various, often conflicting aspects and interests are focused in the lakeshore zone. The most important of these are the settlement process and tourism development, as well as issues related to the protection of both the lakes themselves and their shores. The impact of human activities on the lake environment has been widely described (e.g., [21,24]). Many authors have analyzed lake shoreline development using GIS datasets (e.g., [25,26]). 

The aim of the study was to identify the main trends in land use and land cover changes in the lakeshore zone at the background of socio-economic changes in Poland and Hungary. The focus was on infrastructure for water recreation and tourism, especially sailing tourism.

## 2. Study Area

The study area included areas around lakes located in two former socialist countries that are now members of the European Union—Poland and Hungary. These countries are characterized by similar socio-economic conditions, but at the same time they differ fundamentally in their geographical and natural conditions (size and number of lakes, access to the sea, and climate). We took into account a 100 m strip of land along the shoreline of lakes and canals in both countries. 

### 2.1. Poland

In the Polish part of the study area, the focus was on a complex of 34 lakes connected by 6 canals (total area of the water surface equaled 330 km^2^) in two neighboring mesoregions: Great Mazurian Lakes and Mazurian Plain, belonging to the Mazurian Lakeland macroregion, in north-eastern Poland [27]. This macroregion is one of the major tourist destinations in Poland. It owes its tourist attractiveness to its valuable natural resources, varied, postglacial landscapes and, above all, numerous lakes. 

**Figure 1:**
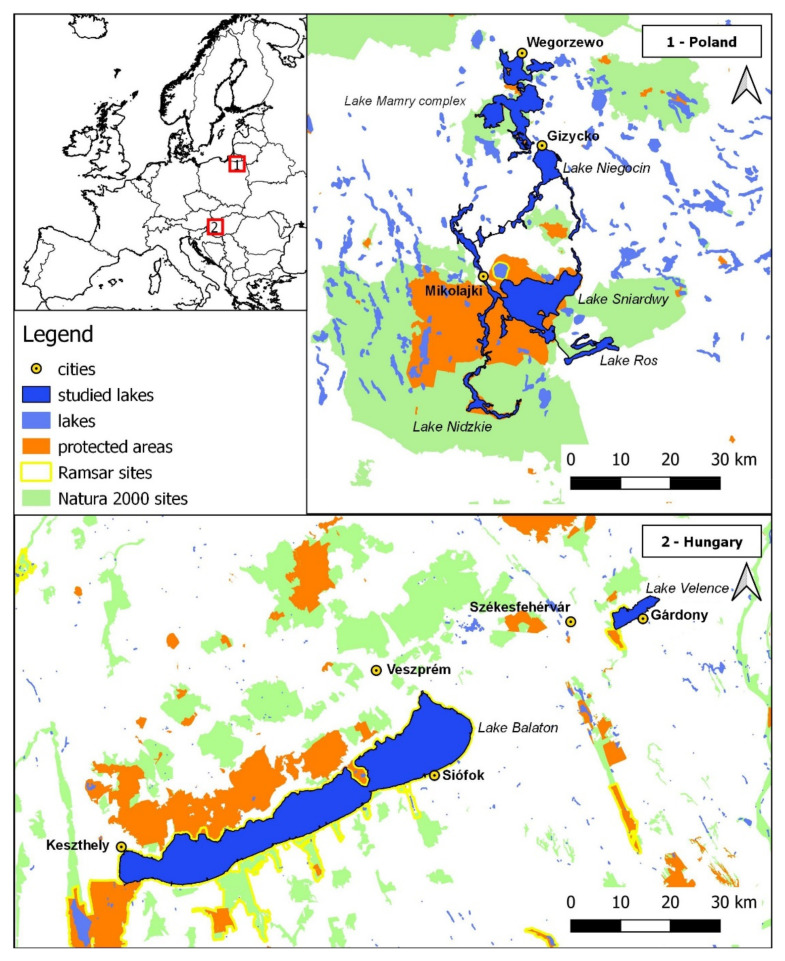
The study area.

The Great Mazurian Lakes region comprises the largest lakes in Poland. Lakes cover more than 20% of the region. The lakes studied are an extensive system of sailing routes appreciated by tourists. The main waterway (from Lake Roś in the south to Lake Mamry in the north) is 110 km long and has numerous branches [28]. The extreme coordinates of the study area are 53°33′ and 54°13′ North latitude and 21°28′ and 22°00′ East longitude. The study area covers 1870 km^2^ in 14 communes of the warmińsko-mazurskie voivodship (Figure 1). The largest city of the region is Giżycko (about 30,000 inhabitants). Numerous post-glacial forms, such as kames, eskers, erratic boulders, occur in the study area. The studied area is free from industrial damages, especially those of heavy and mining industries. Protected areas of international importance, belonging to the Natura 2000 network, constitute about 51% of the study area (Figure 1). The size of the lakes studied ranged from 0.2 km^2^ to 113.8 km^2^, with maximum depths ranging from 3.2 m to 50.8 m and trophic status from mesotrophic to eutrophic.

### 2.2. Hungary

In the Hungarian part of the study area, the focus was on two large natural lakes: Lake Balaton and Lake Velence. Lake Velence is situated in the Central Transdanubian region, Lake Balaton is on the border of the Central and the Southern Transdanubian Region, in the western countryside (Figure 1). Lake Balaton is one of the most popular holiday destinations in Hungary: the south banks are full of modern beach resort, the northern banks provide old villages and historical sites, as well as some wineries. Lake Velence has a similar duality, both with nature values and its tourist and recreational facilities. By the 1960s, large scale bank protection works and lake basin control works carried out on both lakes, mainly based on placing the rip-raps, which had fundamentally changed the natural characteristics and ecology of the lakes’ shore zone (littoral and riparian zone), often followed by algae blooms [29]. In the last two decades Lake Balaton has a rather mesotrophic [30], Lake Velence shows an eutrophic character. 

Lake Balaton is the largest lake in Hungary, and the largest freshwater lake in Central Europe, the surface area is 596 km^2^, a mean depth of 3.2 m, and a catchment area of 5775 km^2^ [31]. The whole lake basin belongs to the Natura 2000 network, same northern shore sections connected to the Balaton Uplands National Park [31]. The largest city by the lake is Siófok (24,800 inhabitants). Lake Velence is one of the largest Hungarian shallow lakes with a surface area of 24.17 km^2^, the average depth is 1.45 m, the catchment area is 602.4 km^2^ [32]. The western basin is covered by emergent macrophytes, the eastern basin is dominated by the open water-surfaces. On the western part of the lake a nature conservation area of 4.2 km^2^ is situated belonging to the competence of Ramsar Convention, also a part of the Natura 2000 network. The largest city by the lake is Gárdony (11,000 inhabitants).

## 3. Materials and Methods

Land use and land cover (LU/LC) maps were prepared using topographic maps and orthophotograph maps scaled 1:10,000. The vector polygon database was created based on digitized raster orthophotograph maps dated 2016–2018, current vector cadastral maps (geoportal.gov.pl and private resource) and raster digitized topographic maps dated 1989. To ensure that types of LU/LC were correctly classified prepared maps were verified in the field. Polish raster maps were obtained from Provincial Centre for Geodetic and Cartographic Documentation in Olsztyn, Poland (license number IG-WODGiK.7522.146. 2019_28_N). Hungarian raster maps were obtained from Bing aerial and Google satellite map. The total area of analyzed land was 65 km^2^ at the Polish and 25 km^2^ at the Hungarian part of the study area. The studied strip of land was divided to 65 sections in Polish and 25 sections in Hungarian part of the study area. These sections were approximately 1 km^2^ in area (10 km long and 100 m wide) and were the primary study fields. The initial stage of the study was to use the CORINE Land Cover classification to categorize the land in the shore zone of the lakes [33]. The second step was to manually verify and precisely correct the initial CORINE Land Cover classification to information from orthophotograph and topographic maps scaled 1:10,000 with the help of QGIS 3.10 software. These verification was provided separately on the maps from 1989 and from 2016–2018. The five classes of land use and land cover were identified (Table 1). 

The third step was to distinguish the areas occupied by tourism development from the whole “developed” areas. These were initially designated using Google Maps, Open Street Map and information obtained from regional tourism organizations. Facilities related to boating such as marinas and harbors were selected and counted. All preliminary information concerning LU/LC types was finally verified and completed by direct field studies in summer–autumn season (June–October) of 2020. The annual rates of LU/LC change were calculated for each class following the model of FAO forest change assessment described in Equation [34]:

q = [(A_2_/A_1_)^1/(t^_2_^−t^_1_^)^ − 1)] × 100%

where q is the annual rate of LUCC in the study area; A_1_ and A_2_ represent the area of LU/LC of interest at the times t_1_ and t_2_ (at the beginning and at the end of the study period).

The Spearman’s rank correlation coefficient was used to check the presence of correlation between the level of development of the lakeshore zone and also to determine the correlation between LU/LC changes in the studied period. The Wilcoxon signed-rank test was used to determine statistically significant differences in LU/LC between the time slices studied. The t-Student test was used to determine statistically significant differences between studied countries in particular time slices.

## 4. Results

**Figure 2:**
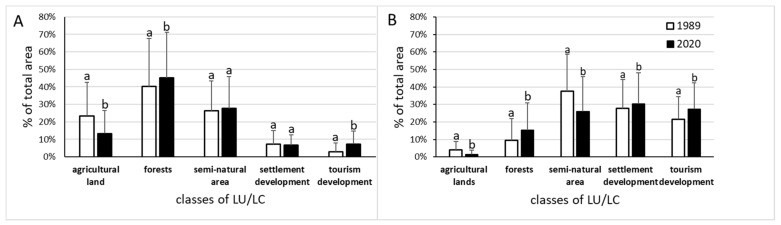
Changes in land use and land cover (LU/LC) in the period of 1989–2020 in Poland (**A**) and Hungary (**B**). Data marked with the same letter (a or b) did not differ statistically (*p* < 0.01).

Detailed changes in LU/LC were illustrated at Figure 2. Decline of agricultural land area and afforestation of the shore zone were observed in both studied countries, as well as tourism development area increase. Changes of the semi-natural land and settlement development areas were significant only in Hungarian lakes’ shores. 

**Figure 3:**
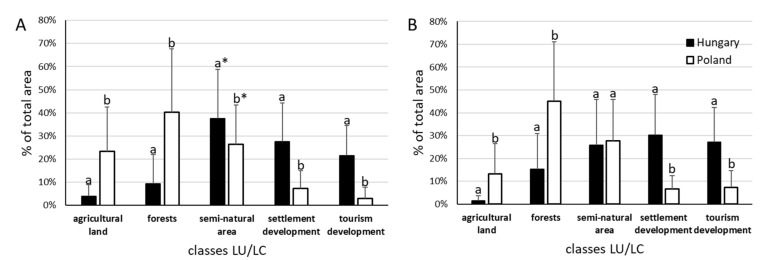
The comparison of land use and cover (LU/LC) in Poland and Hungary in the period of 1989–2020 ((**A**,**B**), respectively). Data marked with the same letter (a or b) did not differ statistically (*p* < 0.01, * *p* < 0.05).

The most important increase of the land coverage (%) was noted for tourism development in Polish lakes’ shores, whereas in Hungarian lakes’ shores the greatest increase was observed for the forests (Table 2). The difference between the studied time slices in the Great Mazurian Lakes was not statistically significant for semi-natural land and for settlement development. For the other LU/LC types, the difference remained statistically significant (*p* < 0.01). For settlement development, this result was unexpected. Especially, that in the shore zone of the Hungarian lakes for all land use and land cover types statistically significant (*p* < 0.01) differences between time slices were shown. A comparison of the study area in both countries shows that in 1989, the share of area occupied by different land use and land cover types in the lakeshore zone showed statistically significant differences (Figure 3A). In the Hungarian part of the study area, the share of land occupied by developments (both residential and tourism) was several times higher than in the Polish part, while the share of land occupied by farmland and forests was several times lower. The smallest differences were observed in relation to semi-natural areas (significance at the level of *p* < 0.05). In 2020, the status from 1989 changed significantly only for semi-natural areas (Figure 3B). The share of area covered by this land type in the lake shore zone in Poland and Hungary almost equaled. For the other land use and land cover types in the lake shore zone in both countries, the situation did not change (Figure 3). Total “developed” areas share in the shore zone of Polish and Hungarian lakes differs significantly (10.23% vs. 49.1% in 1989 and 14% vs. 57.4% in 2020, respectively). 

**Figure 4:**
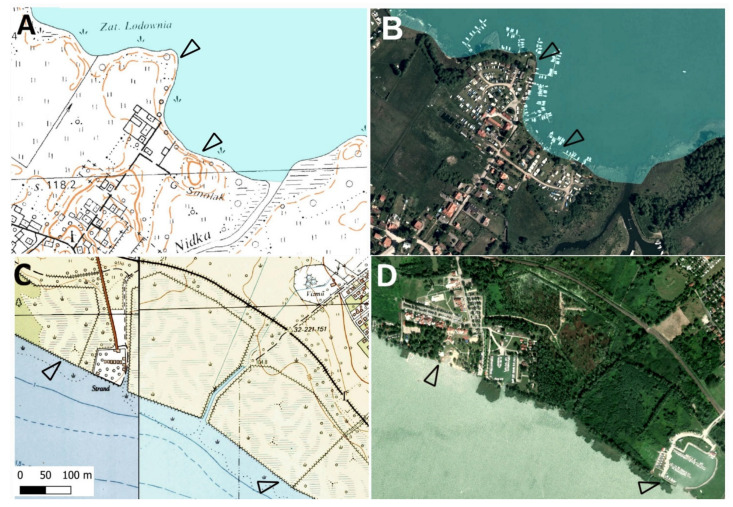
The land use and land cover changes of the lakeshore zone—the examples of: Lake Bełdany (53°41′08″ N 21°33′07″ E: (**A**,**B**)) and Lake Balaton (46°45′42.5″ N 17°17′22.3″ E: (**C**,**D**)). Arrowheads indicate (respectively) farmland in 1989 (**A**) and semi natural land in 1989 (**C**) transformed into tourism development area in 2020 (**B**,**D**).

**Figure 5:**
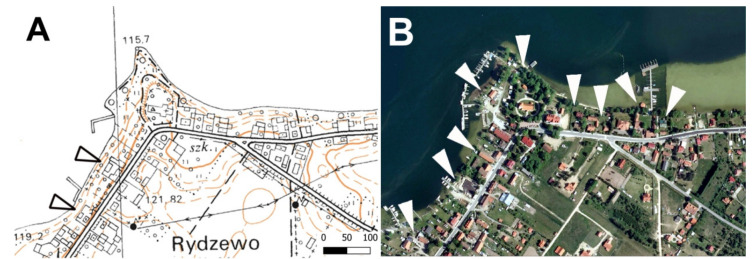
Tourism development (marked by arrowheads) at the shore zone of the Lake Niegocin, Rydzewo village (53°57′55″ N 21°45′36″ E) in 1989 (**A**) and 2020 (**B**).

A significant increase in the area of tourism development in the lake shore zone was observed in both studied countries (Figure 2, Figure 3, Figure 4 and Figure 5). The development of tourism and water recreation (such as boating, sailing, yachting) contributed to the intensive development of marinas and harbors as well as recreationally developed areas. Many sailing schools and yacht clubs were also established on the lakes in the studied period. In 2021, there were a total of 60 marinas and 29 harbors on Lake Balaton and Lake Velence, of which 49 on Lake Balaton and the remaining 11 on Lake Velence. Seven of them had a capacity of more than 200 berths. The marinas on Lake Balaton were mainly concentrated around Keszthely and Balatonfüred, while those on Velence were located on the southern shore of the lake. In the 1980s, there were 18 and 10 marinas on the Balaton and Velence lakes, respectively. In 2021, there were 138 marinas on the Great Mazurian Lakes with a total area of more than 90 ha and a capacity of more than 3000 berths. Most of them were located in the region of Giżycko and Mikołajki. In the 1980s, there were just 37 marinas. In the Polish part of the study area, according to Spearman’s rank test, a negative correlation was found between changes in the area of agricultural land and changes in the area occupied by settlement development (*p* < 0.01, r_S_ = −0.32). A similar situation was observed for forests and semi-natural land (*p* < 0.01, r_S_ = −0.36). The strongest negative correlation was observed for agricultural land and semi-natural land (*p* < 0.01, r_S_ = −0.68). The only positive correlation was observed between the area of tourism development and the area of settlement development in 2020 (*p* < 0.01, r_S_ = 0.47). In the Hungarian part of the study area, according to Spearman’s rank test, a negative correlation was found between changes in the area of agricultural land and forests (*p* < 0.01, r_S_ = −0.4) and between changes in the area of semi-natural land and forests (*p* < 0.01, r_S_ = −0.54).

## 5. Discussion

The most important trend in land use change was an increase in the area occupied by tourism development and forests at the expense of agricultural land (Poland) and semi-natural areas (Hungary). Hungary has also seen an increase in settlements. According to Cegielska et al., the area of built-up land, forests and semi-natural land increased at the expense of agricultural land in both countries between 2000 and 2012 [35]. Li et al. showed that in the former USSR there was an increase in the area of built-up land at the expense of agricultural land and forests [26]. Changes in land use in the Great Mazurian Lakes shore zone are probably the result of two parallel processes. On the one hand, it is an effect of the desire to maximize profits from land use; on the other hand, it is an effect of intensification of environmental protection measures. According to Bičík et al., the conversion of arable land around lakes into other types of land is economically viable and beneficial for the natural environment [36]. Tourist and recreational use of these attractive areas is more profitable than agriculture.

The development of real estate and tourism accommodations is notable in the Hungary Lake region (primarily in Balaton Lake). The fragmentation of the shoreland into a large number of small properties after privatization and profit intention lead to the abandonment of agricultural uses and the increase of build-up area [37]. In the shore zone of the Hungarian lakes, land use changes had partly the same reason as in Poland: maximizing profit on attractive lakeshore lands. On the other hand, the decrease of agriculture land use is mainly a result of the changed lifestyle: younger generations prefer urban lifestyle, looking for a livelihood in the service and industrial sector, than in agriculture. After 1990 the number of tourists decreased on Lake Balaton, which together with the decline of agriculture, fertilization and livestock farms, was a positive event for the ecological status of Lake Balaton, resulting improvement in water quality [38]. Since 2001, the annual number of guests in the Lake Balaton region has been gradually increasing, except for the decrease after 2008, which means an increase of nearly 800,000 between 2001–2018. After the latest reform of the Common Agricultural Policy, the agri-environmental payments are also significant considering their effect on land use and their territorial coverage, e.g., by subsidizing non-productive investments aiming at the development [17,39]. 

Replacing agricultural land by built-up areas or forests is a main trend of LU/LC change in Europe [40]. Kuemmerle et al. noted a clear East–West divide in Europe with respect to agriculture, with larger declines in farmland and lower crop intensity in the East compared to the West [41]. These changes are driven by the desire to increase returns from land use [42]. Agricultural use of fragile ecosystems has a negative impact on them [43] and therefore, the above mentioned changes are beneficial for the natural environment. As a result of intensive farming, significant amounts of nitrogen and phosphorus compounds enter into surface waters from agricultural areas. This phenomenon significantly accelerates the process of eutrophication of lakes. The conversion of agricultural land in catchments to other land uses results in a reduction of the nutrient load flowing into waters [44], which leads to an improvement in the condition of lakes [45]. The reduction of agricultural land and the increase of forest area around the Great Mazurian Lakes and the Hungarian lakes Balaton and Velence were beneficial for the ecosystems of these lakes. Kertész et al. found that the conversion of agricultural land to forest in the Balaton catchment was beneficial for ecosystem services [46]. Replacing intensive cropping with forest or semi-natural areas leads to the restoration of ecosystems [47,48]. Additionally, urbanized areas in the shore zone can have a beneficial effect on the lake ecosystem as they reduce the catchment area of the lake. However, upgrading the sewage disposal system is crucial to reduce the nutrient load flowing into lakes [49]. According to Schneider et al. CORINE land cover may be more useful for characterizing littoral nutrient enrichment than lake water chemistry analysis [50]. Sanchez et al. noted that LU/LC had greater influence on phytoplankton morpho-functional groups than physical and chemical variables [51].

In the shore zone of the Polish lakes, there has been a significant increase in the area occupied by tourism development at the expense of agricultural land. On the Hungarian lakes, this process took place at the expense of semi-natural areas. Moreover, the share of settlements in the Hungarian lakes’ shore zone increased, also at the expense of semi-natural land. However, the growth rate of tourism development was higher than that of settlement development. No settlement expansion was recorded at the Polish lakes. This situation was the opposite of that in the adjacent Olsztyn Lakeland [52]. The increase of tourist infrastructure connected with sailing (marinas and harbors) in warmińsko-mazurskie voivodship was noted [53,54]. The number of companies offering yacht charters on the Great Mazurian Lakes in 2017 was twice as high as on the Polish Baltic coast [55]. In Hungary, the fastest growing and popular branches of tourism in Lake Balaton are angling and yachting tourism. The number of yacht places increased to 12,000 by the construction between 2002 and 2012 [38]. 

In the lakeshore zone of Hungary, the growth of tourism development land is mainly reflected in the increase of recreational areas, commercial services established (e.g., a large shopping center was built in Velence bay), and newly constructed marine and yacht harbor. In addition, it is necessary to point out that tourism development and land use change also affect the water surface area and shorelines in the Hungarian studied lakes. Some of the nearshore water areas in both lakes have been decreased from 1986 to 2019 since some of the water areas were transform into beaches or recreational areas in Lake Balaton, and some segments in Lake Velence were infilled by semi-natural land and aquatic plants. In Hungary and by the shores of Hungarian lakes, the key driver of migration into cities—and therefore the expansion of settlements—is the demand for a new lifestyle. The increase in the proportion of developed land is a result of the expansion of residential and commercial areas. Despite the declining population, areas not used for agriculture (including settlements) continue to grow [17]. The population’s growing need for housing and the need to create new employment leads to the transformation and shifting of existing settlement edges.

The positive correlation between settlement development and tourism development area in the lakeshore zone of Polish lakes indicates that tourist facilities were located near pre-existing settlement development. The area of the settlement development not changed significantly. Some of the new tourist facilities had been created at the expense of former settlement buildings. Most of the villages located in the shore zone of the Great Mazurian Lakes are now of a strictly tourist character. This phenomenon confirms that the spatial development policy in the Great Mazurian Lakes region is properly executed. It considers both the interests of local communities and tourists, as well as the requirements of lake ecosystem protection. The expansion of settlements and tourist facilities were separated processes at Hungarian lakes in some periods. After the Second World War, mostly in the 1960s and 1970s, new tourist facilities (boat harbors, beaches, cabins, etc.) started to develop at the shores of large lakes—especially at Lake Balaton and Lake Velence [56]. These developments effected those shore sections, which were near natural at that time, relatively far from the traditional settlements, and have become accessible after the shore engineering processes. After the 1990s, as a result of settlement expansion processes (described above), tourism development areas and typical settlement areas merged, and formed a dense, compact developed area, mostly on the southern shores of the large lakes. 

The dynamics of agricultural area decline in Poland and Hungary, as in other post-socialist countries, was higher than in western countries [41]. However, in the lakeshore zone the decline was even higher. Growing developed areas et the expense of semi-natural land in the shore zone seems to be a great danger for the Hungarian lakes. It is expected that lake shore tourism development will continue to increase. As the transport accessibility of the Great Mazurian Lakes region improves due to the construction of the Warsaw-Gdansk (S-7) and the Olsztyn-Augustów (no 16) expressways, the tourist potential in the region will grow. The socio-economic development strategy effectively reconciles the interests of the tourism sector with the requirements of lake environment protection. A sustainable land use policy in the region prevents the expansion of settlements in the lake shore zone and leads to the concentration of the developing tourism infrastructure (and thus tourist traffic) in already existing localities [57]. Land use conflicts and environmental problems in lakeshore zones could be largely resolved by creating appropriate regulations that include spatial and temporal limits on recreational activities permitted in the riparian and littoral zones. The buffer zone of the Hungarian lakes has great tourism and economical potentials, however, the sides of ecological and aesthetic values cannot be ignored. 

**Figure 6:**
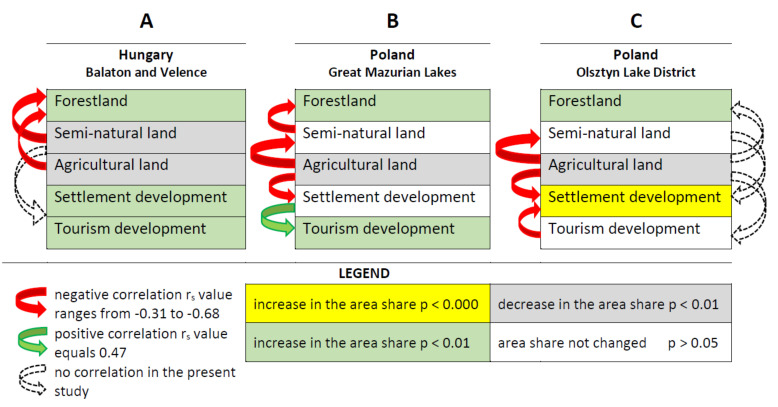
Scheme of different LUCC trends in lake shores: (**A**)—built-up expansion, (**B**)—sustainable tourism development, (**C**)—huge settlement expansion ((**C**)—based on data published in [52]).

LU/LC changes in the Great Mazurian Lakes shore zone may show opposite trends even in adjacent regions. Such a situation was reported in the Mazurian Lakeland ([52] vs. present study). A different pattern of LU/LC changes was observed over the Hungarian lakes (Figure 6). These differences are probably due to the specificity of the studied lakeshore areas. Lakes Balaton and Velence in Hungary are the main natural tourist attraction in the country. In Poland, the Mazurian Lakeland is one of the three (next to the mountains and the Baltic coast) main natural tourist attractions. However, this applies mainly to the Great Mazurian Lakes—a water complex allowing to practice qualified tourism, such as sailing. Settlement pressure threatening the shores of lakes around the city of Olsztyn (described by Furgała- Selezniow et al. [52]) does not occur on the shores of lakes belonging to this complex. Future studies in the field should focus on the economic and social impacts of land use changes around lakes. Another important direction for further research is the impact of changes in land use and land cover in lake catchments on the health of lake ecosystems. 

## 6. Recommendations

Conservation of undeveloped areas in riparian and littoral zones to maintain the unique character of these areas as well as to protect the lakes themselves requires clear legal rules. On some stretches of shorelines, where conditions are undisturbed, priority should be given to nature conservation (with limited access). Increasing the share of public spaces is also of great importance, therefore the maintenance and design of shores with free access should be aimed at so that their spatial distribution is as even as possible. Regular assessment and monitoring is required. It is necessary to create an integrated data system based on open map resources.

We recommend that local authorities of the Great Mazurian Lakes region continue the sustainable spatial development policy in the lake shore area. In particular, it consists of replacing agricultural crops with forests and concentrating tourist facilities within the borders of existing villages. Appropriate legal regulations, which effectively block settlement expansion in the buffer zone around the lake shoreline, are also crucial.

For the Hungarian lakes, in order to use the lake shores sustainably and wisely, the future development of this zone should be based on a comprehensive plan, integrating both ecological and long-term social aspects, complemented by an environmental education program on the ecosystem services of the lakes and lake shores. Urban development is not recommended in lake riparian zones: if necessary to achieve this goal, local plans, building code, zoning system of villages along lake shores should be modified. The required width of this undeveloped buffer zone should be determined based on the characteristics of the shoreline section in question. Regulations should make it clear that the undeveloped zone should never be narrower than 50 m.

## 7. Conclusions

1. Significant increases in the area of tourist development and forests in the lakeshore zone were noted in both countries studied.
2. The conversion of agricultural land to forest and built-up areas is beneficial for the lakes.
3. Settlement expansion is not a real threat to the shores of Mazurian Great Lakes. Appropriate legislation effectively constraints this negative process.
4. Tourism development on the Great Mazurian Lakes is concentrated in towns and villages in correlation with their settlement function.
5. Increasing built-up area at the expense of the semi-natural land seems to be the main threat to the shores of Hungarian lakes.
6. Growth in the area occupied by tourism and settlement development in Hungarian lakes’ shore zone is independent and occurs at different rates.
7. The intensive use of recreational land and the continuous development of entertainment offers (such as water recreation and yacht tourism) in the Hungarian lakes’ shore zone have brought great pressure to the lakes.

## Figures and Tables

**Table 1:** Land use and land cover (LU/LC) classification system.

Class	Corine Code	
Settlement development	111, 112, 121, 122, 133	developed
Tourism development	141, 142 (and 111, 112 after verification)	
Forests	311, 312, 313	undeveloped
Agricultural land	211, 231, 241, 242,	
Semi-natural land	243, 244, 321, 322, 324, 411, 512	

**Table 2:** The magnitude of changes of particular classes of LU/LC in the period of 1989–2020.

Class	Magnitude of Changes [ha]		Magnitude of Changes [%]		q [%]	
	Poland	Hungary	Poland	Hungary	Poland	Hungary
Settlement development	−39.3	67.2	−8.3	9.6	−0.29	0.31
Tourism development	285.5	149.5	152.9	27.0	3.14	0.80
Forests	313.5	148.4	12.0	61.1	0.38	1.60
Agricultural land	−626.7	−63.8	−42.6	−64.2	−1.72	−3.16
Semi-natural land	64.2	−294.2	3.8	−30.5	0.12	−1.21

## Data Availability

The datasets generated and analyzed for this study can be requested from the corresponding author.
